# Arab nations lagging behind other Middle Eastern countries in biomedical research: a comparative study

**DOI:** 10.1186/1471-2288-9-26

**Published:** 2009-04-17

**Authors:** Hani TS Benamer, Omran Bakoush

**Affiliations:** 1 Department of Neurology, New Cross Hospital, Wolverhampton, UK; 2 Department of Nephrology, Clinical Sciences, Lund University, Lund, Sweden

## Abstract

**Background:**

Analysis of biomedical research and publications in a country or group of countries is used to monitor research progress and trends. This study aims to assess the performance of biomedical research in the Arab world during 2001–2005 and to compare it with other Middle Eastern non-Arab countries.

**Methods:**

PubMed and Science Citation Index Expanded (SCI-expanded) were searched systematically for the original biomedical research publications and their citation frequencies of 16 Arab nations and three non-Arab Middle Eastern countries (Iran, Israel and Turkey), all of which are classified as middle or high income countries.

**Results:**

The 16 Arab countries together have 5775 and 14,374 original research articles listed by PubMed and SCI-expanded, respectively, significantly less (p < 0.001) than the other three Middle Eastern countries (25,643 and 49,110). The Arab countries also scored less when the data were normalized to population, gross domestic product (GDP), and GDP/capita. The publications from the Arab countries also have a significantly lower (p < 0.001) citation frequency.

**Conclusion:**

The Arab world is producing fewer biomedical publications of lower quality than other Middle Eastern countries. Studies are needed to clarify the causes and to propose strategies to improve the biomedical research status in Arab countries.

## Background

The quality and the quantity of biomedical publications is used to asses the scientific activities of universities and research centres in individual countries [1], groups of countries [2,3], and even continents [4]. Such analyses make use of scientific bibliographic databases, e.g. PubMed and Science Citation Index Expanded (SCI-expanded). Pubmed is freely available http://www.ncbi.nlm.nih.gov/sites/entrez and widely used. It contains records from > 5000 biomedical journals. SCI-expanded http://scientific.thomson.com/products/sci/, on the other hand, contains bibliographic information from all fields of science, including biomedical research, and covers about 6650 journals in all field of science, but it requires a subscription.

The contribution of the Islamic civilization, of which the Arabs were a central component, to medicine in the Middle Ages is well recognised. Arabic was considered the language of sciences and Avicenna's *"The Canon of Medicine" *was considered the main textbook of medicine in Europe for many centuries. Nowadays, the Arab countries are counted among the developing countries and have a combined estimated population of about 315 million. The Arab populations share a language, and most of them share a religion, culture and historical background. Thus, they are frequently regarded as one unit despite the differences in wealth and population size. Over the last four decades, living standards in most Arab countries have risen, especially those with oil-based economies, the number of undergraduate and postgraduate medical institutions have increased, and health services have improved. This should have been accompanied by increases in scientific research output. Qualitative and quantitative evaluation of biomedical research publication in the Arab countries is essential for monitoring and improving this activity.

Published studies assessing biomedical research output of Arab countries have been mainly quantitative [1,2,5,6]. Therefore, this study analyses both the quantity and quality of biomedical publications of original research articles in Arab countries with high or middle income (World Bank classification), and compares the Arab countries with non-Arab Middle Eastern countries of similar income. This could help in putting the publication activities in the Arab countries in perspective.

## Methods

Sixteen Arab countries of high or middle income (World Bank classification) were included: Morocco, Algeria, Tunisia, Libya, Egypt, Sudan, Lebanon, Jordan, Syria, Iraq, Saudi Arabia, Qatar, Kuwait, United Arab Emirates, Oman and Bahrain. Other Arab countries in the region were excluded because of their low income: Yemen, Comoros, Djibouti, Somalia, Eritrea and Mauritania. Iran, Israel and Turkey were included as non-Arab countries in the region.

Two scientific bibliographic databases, PubMed and SCI-expanded, were selected because they implement acceptable international standards. They were searched for the number of "*original research" *articles published between January 1^st^, 2001 and December 31^st^, 2005 and affiliated to the countries being studied. We have chosen to report only original articles because they represent the research activities. All searches were completed within one day on 06^th ^February 2008 to avoid bias due to the daily update of the databases.

Search of PubMed was carried out in two steps:

1- In the search field, the name of the country was queried using the sensitive query described by Tadmouri & Bissar-Tadmouri [7]. For example, for United Arab Emirates we used "United Arab Emirates" OR UAE OR "Emirats Arabes Unis."

2- The limits facility of PubMed was used to obtain only original research articles, as follows: from January 1^st ^2001 to December 31^st ^2005, clinical trial, meta-analysis, randomised controlled trial, clinical trial Phase I, clinical trial Phase II, clinical trial Phase III, clinical trial Phase IV, comparative studies, controlled clinical trial, evaluation studies, multi-centre studies, research support, non U S Gov't, twin studies, validation studies, English, French.

SCI-expanded was searched as follows:

1- SCI-expanded was queried for biomedical research articles affiliated to the afore-mentioned countries. In the advanced search field, SCI sensitive search field tag "cu" was used to search for the name of the country, e.g. cu = Egypt retrieves all the publications affiliated to Egypt.

2- The search was limited to the five-year period from January 1^st ^2001 to December 31^st ^2005.

3- The search was limited to original research articles. Meeting abstracts, reviews, letters, editorials, corrections, reprints, news items, biographical-items and book reviews were excluded.

4- Non-biomedical articles were excluded by screening the types of articles according to subject. Therefore, subjects such as medicine, surgery, paediatrics, pharmacology, physiology, pathology, infectious diseases, parasitology, microbiology, biochemistry and laboratory medicine, molecular and cell biology, genetics, radiology, neurology, ophthalmology, dermatology, orthopaedics and rheumatology, and public health were used. Subjects such as engineering, energy, physics, chemical chemistry, telecommunication, fishing, water, and transportation were excluded.

6- The article types were rechecked according to journal source. This is an automatic function of the database that ensures that all the articles are published in biomedical journals.

7- The database then generates a count of the total number of original articles, the total citations, and the value of the h-index (highly cited index). The six-year impact factor was defined as the total number of citations found on the search date divided by the number of articles being studied. The h-index was originally developed as a measure of qualifying research performance [8,9]. It is defined as the number of items, *h*, which have at least *h *citations (for example: h-index of 12 means that there are 12 items that have 12 citations or more).

The raw results from each country were normalized by the average of that country's population (2000–2005, the population division of the Department of Economic and Social Affairs of the United Nations Secretariat, World Population Prospects, http://esa.un.org/unpp/), and the gross domestic product (GDP) and GDP/capita (2003–2006, World Bank, http://web.worldbank.org/WBSITE/EXTERNAL/DATASTATISTICS/0,,contentMDK:20535285~menuPK:1192694~pagePK:64133150~piPK:64133175~theSitePK:239419,00.html).

Biomedical research performance was assessed by the following criteria:

1- the number of the original research articles indexed in PubMed and SCI;

2- the h-index;

3- the six-year impact factor (citations frequency);

4- the number of publications in the top medical journals (impact factor >15 according to Science Edition of the Journal Citation Reports, year 2007). The top journals in alphabetical order were the following: *Cancer Cell, Cancer Journal for Clinicians, Cell, Cell Metabolism, Genes & Development, Immunity, Journal of the American Medical, Journal of Clinical Investigation, Journal of the National Cancer Institute, Lancet, Nature, Nature Biotechnology, Nature Cell Biology, Nature Genetics, Nature Immunology, Nature Medicine, New England Journal of Medicine*, and *Science*.

Statistical analysis was performed with SPSS 12.0.1 for Windows (SPSS Inc., Chicago, lL, USA). The differences between groups were compared using the non-parametric Mann-Whitney U-test or Chi-square (χ^2^) test, as appropriate. A p-value < 0.05 was considered significant.

## Results

The 16 Arab countries included in the study have a total population of 267.1 million and a GDP of $1082 billion. The other three Middle Eastern countries have 158.2 million inhabitants and a GDP of $749 billion.

According to SCI-expanded, the Arab countries produced 14,374 original biomedical publications during 2001–2005, which is less than 30% of those produced by the other three Middle Eastern countries (49,110 articles); according to PubMed, they produced 5775 articles, which is less than 23% of those produced by the three non-Arab countries (25,634 article) (p < 0.001, Table 1). PubMed indexes the address of only the first author, whereas SCI indexes the addresses of all coauthors. That could explain the difference in the numbers of articles recovered by the two databases.

**Table 1:** The total number of original research articles from 16 Arab countries and three non-Arab Middle Eastern countries published in PubMed and SCI-expanded during 2001–2005 and their citations frequencies (impact factors).

Country	Number of articles in			Impact factor*	h-index
	PubMed	SCI	high impact journals		
Iran	1910	2965	5	4.96	31
Israel	13637	20504	369	11.89	110
Turkey	10087	25641	38	4.5	56
**Total**	**25643**	**49110**	**412**	7.1**	

Algeria	84	254	5	9.19	21
Bahrain	51	158	0	2.61	8
Egypt	1180	3214	9	5.78	37
Iraq	64	138	3	3.84	11
Jordan	464	822	5	3.7	20
Kuwait	632	1207	8	4.78	24
Lebanon	447	965	7	6.14	31
Libya	19	87	0	3.18	9
Morocco	346	1267	6	3.74	23
Oman	134	436	3	3.43	14
Qatar	50	166	0	3.2	9
Saudi Arabia	999	3134	22	4.26	38
Syria	58	130	0	6.01	13
Sudan	134	265	3	7.18	18
Tunisia	592	1376	9	4.77	26
UAE***	521	755	3	5.93	26
**Total**	**5775**	**14374**	**83**	4.9**	

* Six-year impact factor, 2002–2007

**Average

***UAE: United Arab Emirates

**Figure 1:**
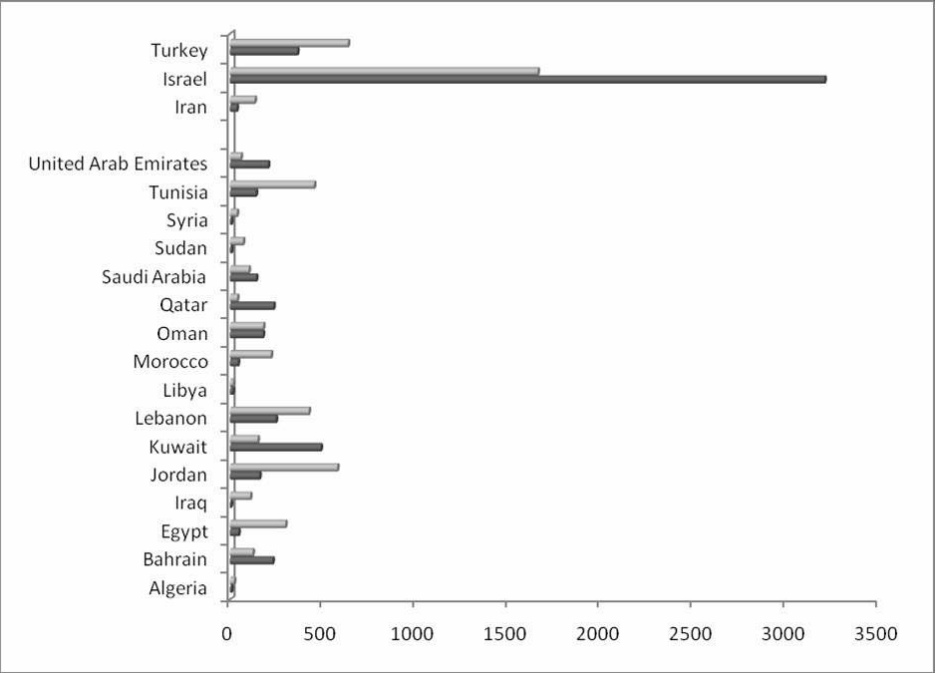
**The total number of original biomedical research articles indexed in SCI-Expanded and affiliated to 16 Arab nations and three non-Arab Middle Eastern countries per million population (dark grey) and per $100 million GDP (light grey)**.

**Figure 2:**
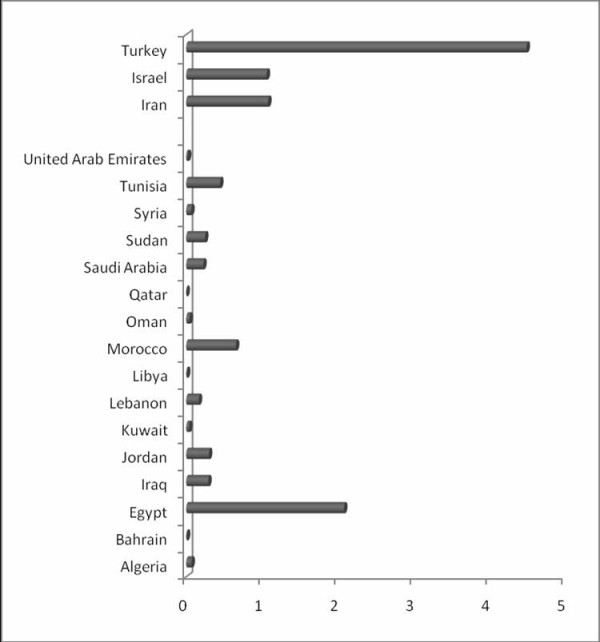
**The number of SCI-indexed original biomedical research publications from 16 Arab and three non-Arab Middle Eastern countries per GPD/capita (US $)**.

The Arab countries also have a significantly lower publication rate than the other Middle Eastern countries when the number of publications is normalized to population, GDP, and GDP/capita (Table 2, Figure 1, 2). Jordan topped the Arab countries when the data were normalized to GDP, and Kuwait came first when data were normalized to population. Egypt ranked first when the number of publications was normalized to GDP/capita (Figure 2).

**Table 2:** Comparison of original biomedical research performance in 16 Arab countries and three non-Arab Middle Eastern countries (2001–2005)

	Countries		P-Value
	Arab	Middle Eastern	
Number of articles according to SCI-expanded	14374.0	49110.0	< 0.001
Number of articles according to PubMed	5775.0	25634.0	< 0.001
Number of publications in the high-impact journals	83.0	412.0	< 0.001
Annual publication rate per one million population	10.8	62.1	0.029
Annual publication rate per one billion (US$) GPD	2.7	13.1	0.001
Annual publication rate per GPD/capita	0.7	2.1	< 0.001

The number of publications in top medical journals, citation frequency (six-year impact factor), and the h-index were all significantly higher in the three Middle Eastern countries (p < 0.001) (Table 1, 2).

## Discussion

We compared the biomedical research performance in the Arab world with that in non-Arab Middle Eastern countries. We show that Arab countries are lagging behind in the number of original biomedical research publications, publications in top medical journals, and citation frequencies (six-year impact factor and h-index). They also lag behind when the number of publications is normalized to population, GDP, and GDP/capita.

Several studies discussed the reasons leading to the paucity of medical research and biomedical publications in the Arab world. Tadmouri & Bissar-Tadmouri [2] suggested that the regional conflicts have been a major reason for the stagnation of medical publications in Arab countries. However, the other Middle Eastern countries have also been exposed to considerable instability and regional conflicts. Lack of freedom, democracy and funding, as well as brain drain and the difficulty of publishing research of local interest in high impact journals, all contribute to the low performance of biomedical research in the Arab world [10,11]. All these factors have to be taken into consideration if the governments of the Arab countries wish to improve the status of their biomedical research.

Previous studies addressed the issue of biomedical publications in the Arab world as a whole or in individual countries [1-3,5,6]. Shaban and Abu-Zidan reported that between 1987 and 2001 Egypt and Saudi Arabia had the most biomedical publications among the Arab countries [6]. Our results are similar, as these two countries had the highest number of original research publications in the Arab world. Also, Kuwait was reported to have the largest number of publications when normalized to the population, and Jordan came first when its data were normalized to GDP [6]. Here we report similar findings, but add that Egypt ranked first in the rate of publications per GDP/capita for 2001–2005, which is in line with the results of Tadmouri & Bissar-Tadmouri for 1988 and 2002 [2]. Therefore, we conclude that Egypt, Kuwait, Jordan and Saudi Arabia are leading the Arab world in biomedical research.

Some studies have indicated that raw counts of publications can be misleading and that counts should be normalized to indicators such as population and GDP [6]. Other studies point out that GDP-normalized bibliometric data could also be misleading, especially in the case of developing countries [2]. Some authors advocate the use of citations as an indication of the quality of research performance [5,12], but others indicated the shortfalls of such an approach [13,14]. Also, false-positive and false-negative results are unavoidable when using scientific bibliographic databases [15,16]. We tried to overcome some of these problems when assessing the quantity and quality of biomedical research publications by specifically selecting the original research articles indexed in two highly prestigious databases (SCI and PubMed) and presenting the data both in raw form and after normalization, as well as using two indicators of citation frequency (six-year impact factor and the h-index). Furthermore, we also counted original research articles published in high-impact biomedical journals as a different indicator of research performance.

Nevertheless, it is important to stress that one shortcoming of comparing the Arab countries with other Middle Eastern countries is that research resources are usually more effectively used when they are pooled in a single country rather than divided among many. Therefore, this study has the limitations that are inherent in the different bibliometric methods, and the results should be interpreted in this context. Despite these limitations, our study clearly highlights the modesty of biomedical research performance in the Arab world.

Citation frequency is an important tool in the evaluation of research performance, as it correlates with the peer-evaluation of research impact and quality [8,9]. The impact factor and h-index make it easier to gain more information about the publications and trends of individuals, institutes, and countries. Arab countries should pay attention to the low citation frequencies of their research publications. Medical schools and research institutes have to set short and long term plans to improve the impact of the local research. Arab countries should benefit from the experience of other countries that have equivalent economic resources but outperform them in biomedical research.

## Conclusion

This study shows that Arab countries are not producing enough biomedical publications compared to other Middle Eastern countries with similar economic conditions. Action is necessary to improve the status of biomedical research in Arab countries. Studies are needed to clarify the causes and to propose strategies to improve the biomedical research status in the Arab world.

## Competing interests

The authors declare that they have no competing interests.

## Authors' contributions

HTSB participated in the design of the study, collected the data from PubMed, and drafted the manuscript. OB participated in the design of the study, collecting data from SCI-expanded, and conducting the statistical analysis, and helped in drafting the manuscript. Both authors read and approved the final manuscript.

## Pre-publication history

The pre-publication history for this paper can be accessed here:

http://www.biomedcentral.com/1471-2288/9/26/prepub

## References

[B1] Tadmouri GO Bissar-Tadmouri N Biomedical research in the Kingdom of Saudi Arabia (1982–2000) Saudi Med J 2002 23 20 24 11938358

[B2] Tadmouri GO Bissar-Tadmouri N Biomedical publications in an unstable region: the Arab world, 1988–2002 Lancet 2003 362 1766 10.1016/S0140-6736(03)14868-4 14643139

[B3] Bakoush O Al-Tubuly AA Ashammakhi N Elkhammas EA PubMed Medical Publications From Libya Libyan J Med 2007 2 125 128 http://2657.indexcopernicus.com/fulltxt.php?ICID=870338 10.4176/070625 PMC3078204 21503210

[B4] Hefler L Tempfer C Kainz C Geography of biomedical publications in the European Union, 1990–98 Lancet 1999 353 1856 10.1016/S0140-6736(99)01278-7 10359422

[B5] Deleu D Northway MG Hanssens Y Geographical distribution of biomedical publications from the Gulf Corporation Council countries Saudi Med J 2001 22 10 12 11255602

[B6] Shaban SF Abu-Zidan FM A quantitative analysis of medical publications from Arab countries Saudi Med J 2003 24 294 296 12704508

[B7] Tadmouri GO Bissar-Tadmouri N A major pitfall in the search strategy on PubMed Saudi Med J 2004 25 7 10 14758370

[B8] Cronin B Meho L Using the h-index to rank influential information scientists Journal of the American Society for Information Science and Technology 2006 57 1275 1278 10.1002/asi.20354

[B9] Meho LI Spurgin KM Ranking the research productivity of library and information science faculty and schools: An evaluation of data sources and research methods Journal of the American Society for Information Science and Technology 2005 56 1314 1331 10.1002/asi.20227

[B10] Maziak W Geography of biomedical publications Lancet 2004 363 490 10.1016/S0140-6736(04)15500-1 14962534

[B11] El Ansari W Afifi Soweid RA Jabbour S Geography of biomedical publications Lancet 2004 363 489 490 10.1016/S0140-6736(04)15498-6 14962532

[B12] Garfield E Citation analysis as a tool in journal evaluation Science 1972 178 471 479 10.1126/science.178.4060.471 5079701

[B13] Baylis M Gravenor M Kao R Sprucing up one's impact factor Nature 1999 401 322 10517624 10.1038/43768-c1

[B14] Gallagher EJ Barnaby DP Evidence of methodologic bias in the derivation of Science Citation Index impact factor Ann Emerg Med 1998 31 83 6 10.1016/S0196-0644(98)70286-0 9437347

[B15] Al-Hajeri AA Fedorowicz Z Amin FA Eisinga A The handsearching of 2 medical journals of Bahrain for reports of randomized controlled trials Saudi Med J 2006 27 526 30 16598332

[B16] Al-Hajeri A Al Sayyad J Eisinga A Handsearching the EMHJ for reports of randomized controlled trials by U.K. Cochrane Centre (Bahrain) East Mediterr Health J 2006 12 Suppl 2 S253 S257 17361697

